# Evolutionary and functional characterization of leucoanthocyanidin reductases from *Camellia sinensis*

**DOI:** 10.1007/s00425-017-2771-z

**Published:** 2017-09-08

**Authors:** Peiqiang Wang, Lingjie Zhang, Xiaolan Jiang, Xinlong Dai, Lijuan Xu, Tong Li, Dawei Xing, Yanzhi Li, Mingzhuo Li, Liping Gao, Tao Xia

**Affiliations:** 1 State Key Laboratory of Tea Plant Biology and Utilization, Anhui Agricultural University, Hefei, 230036 Anhui China; 2 School of Life Science, Anhui Agricultural University, 130 West Changjiang Rd, Hefei, 230036 Anhui China

**Keywords:** Catechins, Leucoanthocyanidin reductases, Phylogenetic analysis, Tea plant

## Abstract

**LARs promoted the biosynthesis of catechin monomers and inhibited their polymerization. The accumulation of catechin monomers and polymers was increased by up-regulating the expression of** ***NtLAR*** **and** ***NtANR*** **s in CsMYB5b transgenic tobacco.**

Tea is rich in polyphenolic compounds, and catechins are the major polyphenols in tea. The biosynthesis of polyphenols is closely related to the expression of the *leucoanthocyanidin reductase* (*LAR*) and *anthocyanidin reductase* (*ANR*) genes. In this paper, an evolutionary analysis and functional characterization of three CsLARs were performed. The phylogenetic tree showed that plant LARs could be grouped into three, including gymnosperms, monocotyledons and dicotyledons (clusters I and II). The eighth amino acid residue in a conserved LAR-specific motif is changeable due to a transversion (G → T) and transition (G → C) that occur in the corresponding codon. Therefore, plant LARs can be classified as G-type, A-type and S-type LARs due to this variable amino acid residue. Although (2R, 3S)-*trans*-flavan-3-ols were the products of recombinant CsLARs proteins expressed in *Escherichia coli*, both (2R, 3S)-*trans* and (2R, 3R)-*cis*-flavan-3-ols were detected in tobacco overexpressing CsLARs. However, a butanol/HCl hydrolysis assay indicated that overexpression of the CsLARs caused a decrease in polymerized catechins. A hybridization experiment with CsLARc + AtPAP1 also showed that no polymers other than epicatechin, catechin and glycoside were detected, although the accumulation of anthocyanins was markedly decreased. CsMYB5b promoted the biosynthesis of both flavan-3-ols and proanthocyanidins (PAs). Therefore, LARs promoted the biosynthesis of catechin monomers and inhibited their polymerization. The accumulation of catechin monomers and polymers was increased by up-regulating the expression of the *NtLAR* and *NtANRs* in CsMYB5b transgenic tobacco.

**Electronic supplementary material:**

The online version of this article (doi:10.1007/s00425-017-2771-z) contains supplementary material, which is available to authorized users.

## Introduction

Polyphenolic compounds are a large class of plant secondary metabolites that are ubiquitously present in many plants, and they mainly consist of flavones, flavonols, anthocyanins, isoflavones, condensed tannins (CT; or PAs) and other derivatives (Winkel-Shirley 2001; Tanner et al. 2003; Xie et al. 2003). Different classes of polyphenolic compounds contribute in various ways to plant growth and defense against biotic and abiotic stress, including pathogen infections, ultraviolet radiation and herbivory (Dixon and Sumner 2003; Mellway et al. 2009). Additionally, certain polyphenolic compounds can act as antioxidants and protect humans from cardiovascular disease, diabetes, ultraviolet radiation and obesity (Cos et al. 2004; Nichols and Katiyar 2010; Wang et al. 2013). Flavan-3-ols and PAs greatly contribute to the astringency of tea, wine and certain fruits, such as persimmon (*Diospyros kaki*) (Kennedy et al. 2008; Ma et al. 2010; Liu et al. 2012).

Tea, which is a perennial evergreen woody plant, is rich in polyphenolic compounds. In tea, dissociative flavan-3-ol monomers are mainly distributed in the leaves of the aerial parts of the plant, and polymeric catechins are distributed in the stems and root (Jiang et al. 2013, 2015). Dissociative flavon-3-ols are also called catechins and include (2R, 3S)-*trans*-flavan-3-ols (catechin, C, and gallocatechin, GC) and (2R, 3R)-*cis*-flavan-3-ols (epicatechin, EC; epigallocatechin, EGC; epicatechin gallate, ECG; and epigallocatechin gallate, EGCG) (Qian et al. 2015). Catechins play a crucial role in the dominating astringent taste of tea and possess a number of effects that are beneficial to human health, particularly the galloylated catechins (Chung et al. 2003; Cui et al. 2016).

**Fig. 1:**
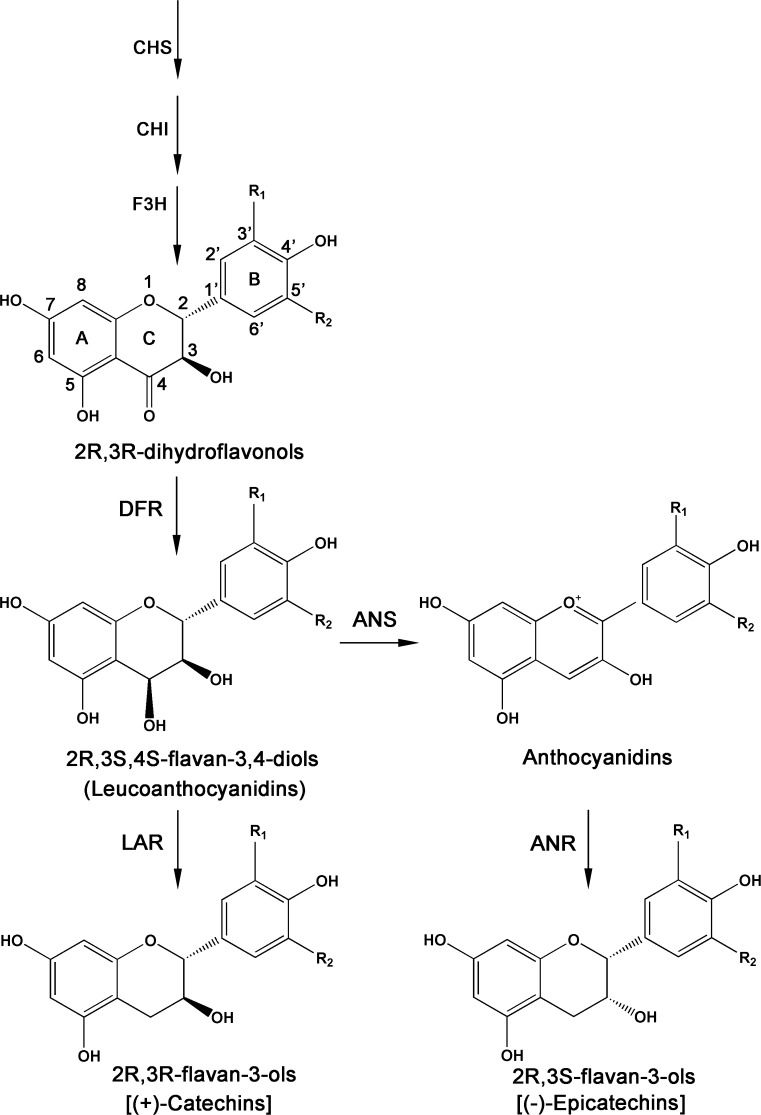
Schematic representation of the biosynthetic pathway of flavan-3-ols

The biosynthesis pathway (Fig. 1) of polyphenolic compounds has been characterized via genetic and biochemical approaches in many plant species, including *Arabidopsis thaliana*, *Vitis vinifera*, *Populus trichocarpa*, *Theobroma cacao* and *Camellia sinensis* (Nesi et al. 2001; Bogs et al. 2007; Li et al. 2012; Liu et al. 2013; Pang et al. 2013). Most structural genes in the flavonoid metabolism pathway have been identified. ANRs catalyze anthocyanins to form epicatechins (epiafzelechin, EFZ; EC; and EGC), and LARs convert the leucoanthocyanidins into the corresponding catechins (afzelechin, AFZ; C; and GC) in vitro (Stafford 1991; Tanner et al. 2003). The biosynthetic pathway of galloylated catechins was shown to be a two-step enzyme reaction in the tea plant (Liu et al. 2012). The functions of two ANRs and one LAR from the tea plant have been identified in previously published papers, but some questions remain (Pang et al. 2013).

Because LARs are essential reductases in the flavonoid pathway, studies have been conducted to investigate LAR enzymes since the 1980s. Stafford and Lester (1982, 1984) first detected the enzyme activity of converting (+)-dihydroquercetin (DHQ) to leucocyanidin and then to (+)-catechin with NADPH as the H-donor in crude protein extracts derived from *Pseudotsuga menziesii*. DuLAR, which was the first LAR protein purified from the PA-rich leaves of *Desmodium uncinatum,* was confirmed to synthesize C, AFZ and GC with 3,4-*cis*-leucoanthocyanidins as substrates by expressing recombinant protein in *Escherichia coli* (*E. coli*), tobacco (*Nicotiana tabacum*) and white clover (*Trifolium repens*) (Tanner et al. 2003). Many *LAR* genes have been identified in various plants, such as *V. vinifera*, *Medicago truncatula* and *Ceratocystis polonica* (Kristiansen 1986; Bogs et al. 2005; Hammerbacher et al. 2014). Subsequently, the crystal structure and catalytic mechanism of VvLAR in vitro were described in detail, which helped us to further understand the function of LARs (Mauge et al. 2010).

Recently, a new discovery in a research study investigating the role of LARs in PA synthesis reported that MtLAR could convert 4β-(*S*-cysteinyl)-epicatechin, a new substrate, into EC in vitro. 4β-(*S*-cysteinyl)-epicatechin, which is also a carbocation form, played an important role in non-enzymatic polymerization and served as a PA extension unit (Liu et al. 2016). However, the source of 4β-(*S*-cysteinyl)-epicatechin is currently unknown.

In this paper, three *CsLARs* in tea plants were cloned and functionally characterized in vivo and in vitro. In addition, the evolutionary relationship between the CsLARs and other plant LARs from gymnosperms and angiosperms is discussed. All of the data suggest that CsLARs are responsible for the biosynthesis of both (2R, 3S)-*trans-* and (2R, 3R)-*cis*-flavan-3-ols in the trangenetic CsLARs tobacco. Co-expression of the *ANR* and *LAR* genes promotes the biosynthesis of both flavan-3-ols and PAs.

## Materials and methods

### Materials

The tea plants (*C. sinensis* cv*. Shuchazao*) used in this experiment were grown in an experimental tea field at Anhui Agricultural University, Hefei, China (East longitude 117.27, North latitude 31.86). Leaves at different developmental stages (buds and 1st, 2nd, 3rd and 4th leaves), mature leaves, tender stems and roots were harvested and immediately frozen in liquid nitrogen for further study. The wild-type *Arabidopsis thaliana* used in our laboratory was ecotype Columbia 0 (Col-0), which was grown in a chamber at a constant temperature of 16 ± 3 °C and a 16/8 h (light/dark) photoperiod. The tobacco (*Nicotiana tabacum* cv. G28) used for the transgenic assays was provided by the University of Science and Technology of China (Hefei, Anhui, China) and grown in a growth chamber at a constant temperature of 24 ± 3 °C and a 12/12 h (light/dark) photoperiod.

*Escherichia coli* DH5α and BL21 (DE3) (TransGen Biotech, Beijing, China) were used as the host strain and expression strain for the prokaryotic expression, respectively. *Agrobacterium tumefaciens* C58C1 and EHA105 were kindly provided by the University of Science and Technology of China.

The substrates DHQ, dihydromyricetin (DHM) and dihydrokaempferol (DHK) and standard C, GC, EC, EGC, ECG, EGCG, procyanidin B2 and cyanidin-3-*O*-glucosides were purchased from Sigma (St. Louis, MO, USA).

### Cloning of *CsLARa, CsLARb, and CsLARc*

Total RNA was extracted from the aforementioned organs of the tea plants using RNAiso mate and RNAiso Plus (Takara, DaLian, China) according to the manufacturer’s instructions. The reverse transcription reaction was carried out using the PrimeScript RT Reagent Kit (Takara). In addition, the 3′ and 5′ RACE (rapid amplification of cDNA ends) Kit (Clontech, Mountain View, CA, USA) was used for the RACE library construction following the manufacturer’s instructions. The full open reading frame (ORF) was amplified by high-fidelity PCR using cDNA from the total RNA. The ORF primers of *LARs* are listed in Suppl. Table S1. The PCR procedure was performed at 98 °C for 30 s, 30 cycles at 98 °C for 10 s, 60 °C for 30 s and 72 °C for 45 s, followed by a final extension at 72 °C for 10 min. The PCR amplification products were gel purified using a Gel Extraction Kit (Aidlab, Beijing, China), ligated into an Easy-Blunt vector (TransGen Biotech) and then transformed into DH5α competent cells for sequencing.

### Expression of CsLARs in *E. coli* and their functional validation

A double gene expression strategy was performed for the functional validation of the CsLARs. The ORFs of *CsLARs* and *CsDFRa* were cloned into the expression vector pRSFduet (Novagen, Carlsbad, CA, USA), which contains two multiple cloning sites (MCS I and MCS II). The primers used for *CsLARs* and *CsDFRa* to construct the protein expression vectors are listed in Suppl. Table S1. The PCR procedure was performed at 98 °C for 30 s, followed by 30 cycles of 98 °C for 10 s, 60 °C for 30 s and 72 °C for 45 s, with a final extension at 72 °C for 10 min. The PCR products and empty pRSFduet vector were digested with the corresponding restriction enzymes (New England Biolabs) at 37 °C for 12 h. After gel purification, the products were ligated into the digested vector using T4 ligase (New England Biolabs) at 16 °C for 12 h before being transformed into DH5α competent cells for sequencing. After sequencing, the empty vector (pRSFduet), pRSFduet–*CsDFRa*, pRSFduet–*CsDFRa* + *CsLARa*, pRSF–*CsDFRa* + *CsLARb* and pRSF–*CsDFR* + *CsLARc* were transformed into expression strain BL21 (DE3) for protein expression. A single colony confirmed by PCR was inoculated into Luria–Bertani (LB) broth containing 50 mg/L kanamycin at 37 °C. When the OD_600_ reached 0.6–0.8, IPTG (isopropyl β-d-1-thiogalactopyranoside) was added to a final concentration of 1 mM to induce protein expression. After the cell cultures were incubated at 28 °C for 6–8 h, dihydroflavonols (DHK, DHQ and DHM), which could be used as substrates by CsDFRa and converted into leucoanthocyanidins, were added to a final concentration of 100 μM. After 8 h, the supernatant of each bacterial suspension was harvested by centrifugation (3500*g* for 15 min at 4 °C) and extracted three times with ethyl acetate. The merged supernatant was concentrated to remove ethyl acetate at a low temperature. Finally, the products were dissolved in 200 µL methanol. To identify the products of DFRa + LARs, reverse-phase HPLC (Shimadzu, Kyoto, Japan) and ultrahigh-performance liquid chromatography coupled to a mass spectrometer (UPLC–MS/MS) (Agilent, Santa Clara, CA, USA) were used as previously described (Cui et al. 2016).

### *Agrobacterium*-mediated transformation of *Arabidopsis* and tobacco

The ORFs of the *CsLARs* were cloned into the Gateway Entry vector pDONR207 using the Gateway BP Enzyme mix according to the manufacturer’s instructions (Invitrogen, Carlsbad, CA, USA). The primers are listed in Suppl. Table S1. After sequencing, the entry vectors were recombined into the plant transformation destination vector pCB2004 using the LR Enzyme mix (Invitrogen). The recombinant pCB2004-LARs were electroporated into *Agrobacterium tumefaciens* EHA105 and C58C1. A single EHA105 colony containing the target construct was confirmed by PCR and inoculated into liquid LB medium containing 50 mg/L kanamycin and 50 mg/L spectinomycin at 28 °C until the OD_600_ of the cell suspension reached 0.6. The *Agrobacterium* cells were collected by centrifugation (3500*g*, 15 min) and used for gene transformation in tobacco following a previously reported method (Fisher and Guiltinan 1995; Wang et al. 2014). Similarly, an *Agrobacterium tumefaciens* C58C1 colony was transferred into *Arabidopsis* using the floral-dip method as previously described (Clough and Bent 1998; Cai et al. 2014).

### Hybridization of different transgenic tobacco lines

Well-grown T1 generation plants of CsLARs transgenic tobacco and AtPAP1 transgenic tobacco were chosen, and their stamens were gently removed before pollination. The pollen of tobacco overexpressing CsLARs and AtPAP1 was then applied to the pistils of AtPAP1 and CsLARs transgenic tobacco, respectively. The pollinated pistils were protected by a transparent bag until the seeds were ripe. The newly grown seedlings were used in subsequent experiments after PCR verification.

### Extraction and quantification of catechins, anthocyanins and PAs

To analyze the catechins in the tea plants, 0.2 g of fresh samples (buds, tender leaves, young stem and young roots) were ground in liquid nitrogen and extracted with an extraction solution (80% methanol and 20% water), followed by vortexing and sonicating for 30 min at a low temperature. Then, the samples were centrifuged at 3500*g* for 15 min, and the residues were re-extracted twice as described above until the final volume of the pooled supernatants was 2 mL. The supernatants were then extracted three times with chloroform and three times with ethyl acetate. The pooled supernatant was concentrated to remove the ethyl acetate at a low temperature with a vacuum pump. Finally, the product was dissolved in 200 µL methanol for further analysis. Six main catechins were quantitatively analyzed following a previously described HPLC method with corresponding standards (Jiang et al. 2013).

To analyze the anthocyanins in the transgenic tobacco flowers, 0.5 g of fresh petals was ground in liquid nitrogen and extracted with an extraction solution (80% methanol, 19.9% water and 0.1% hydrochloric acid, by vol.), followed by vortexing and sonicating for 30 min at a low temperature. Then, the samples were centrifuged at 3500*g* for 15 min, and the residues were re-extracted twice as described above until the final volume of the pooled supernatants was 2 mL. The absorption of the supernatants was measured at 525 nm.

To analyze the PAs in the transgenic tobacco flowers, 0.5 g of fresh petals was ground in liquid nitrogen and extracted with an extraction solution (70% acetone, 29.5% water, and 0.5% acetic acid), followed by vortexing and sonicating for 30 min at a low temperature. Then, the samples were centrifuged at 3500*g* for 15 min, and the residues were re-extracted twice as described above. The pooled supernatants were extracted three times with chloroform and three times with ethyl acetate. The pooled supernatant was steamed to remove the ethyl acetate at a low temperature using a vacuum pump. Finally, the product was dissolved in 200 µL methanol for further analysis.

To detect the soluble PAs in the tobacco flowers, the dimethylaminocinnamaldehyde (DMACA) reagent (0.2% DMACA, w/v) was used as previously described (Li et al. 1996). The absorption of the reaction mixture (DMACA: supernatants = 10:3) was measured at 640 nm. To further identify the product, UPLC coupled to a triple quadrupole mass spectrometer (QQQ-MS) (Agilent) was used following a previously described method (Jiang et al. 2015). A quantitative established method based on the multiple reaction monitoring (MRM) mode of the UPLC–QQQ-MS was used to quantify the products in the different transgenic lines, with EC, C and procyanidin B2 as the standards (Wu et al. 2016).

To extract and detect the soluble PAs from the *Arabidopsis* seeds, the same method was applied, but 50 mg of dry seeds was used as the grinding material. To quantify the total soluble PAs, the DMACA reagent (0.2% DMACA, w/v) was applied using procyanidin B2 as the standard.

To determine the insoluble PAs in the tobacco flowers and seeds from *Arabidopsis*, the residues were dried using a vacuum drier. Then, 1 mL of the butanol–HCl reagent was added to the dried residues, and the mixture was sonicated for 30 min at room temperature, followed by centrifugation at 3500*g* for 15 min. The absorption (A1) of the supernatants was measured at 525 nm to determine the amount of background absorption. After boiling the butanol–HCl reagent and residue mixtures for 1 h at 95 °C, the absorbance (A2) at 525 nm was measured again. The normalized absorbance of the insoluble PAs was calculated by subtracting the A2 values from the A1 values and converting that value into PA equivalents using cyanidin-3-*O*-glucoside as the standard.

### Quantitative real-time PCR

RNA that was extracted from the various tissues was quantified with a spectrophotometer (NANODROP 2000, Thermo Scientific). Reverse transcription of the RNA into cDNA was performed using 2 μL 5 × PrimeScript RT Master Mix (Takara) and 500 ng RNA in a reaction volume of 10 µL. In addition, the cDNA was diluted to 25% (v/v) with deionized water before being used as the template. The quantitative real-time PCR was performed in a reaction mixture volume of 20 µL containing 10 µL SYBR Green PCR Master Mix (Takara), 1.1 µL cDNA and 0.8 µL forward and reverse primer (10 μM). The PCR cycling parameters used were as follows: 95 °C for 30 s and 40 cycles of 95 °C for 5 s, 30 s at 60 °C and 30 s at 72 °C, followed by a melting curve analysis from 55 °C to 95 °C. The transcription abundance was normalized to the transcription abundance of glyceraldehyde-3-phosphate dehydrogenase (GAPDH) and was calculated from three technical replicates. The relative expression level was calculated by a previously published method (Wang et al. 2014).

## Results

### Gene cloning and sequence analysis of the CsLARs

Using reverse genetic approaches, the function of a *CsLAR* from the tea cultivar TRI2043 was verified in previous reports (Pang et al. 2013). At least three transcripts of the *CsLARs* genes were screened against the National Center Biotechnology Information (NCBI, http://www.ncbi.nlm.nih.gov) database by homologous sequence analysis.

In this paper, the full-length cDNA sequences of three *CsLAR*s were obtained from the local cultivar ‘*Shuchazao*’ using the RACE technology. The results showed that the full cDNA lengths of the three *CsLAR*s were 1518, 1401 and 1595 bp with 1029, 984 and 1197 bp open reading frames (ORFs), respectively. Their translated proteins were 37, 36 and 43 kDa, and the predicted pI values were 5.43, 5.24 and 5.27, respectively. The three *CsLARs* genes were named *CsLARa, CsLARb* and *CsLARc* and uploaded to NCBI (GenBank accession numbers are KY615698, KY615700 and KY615699, respectively).

**Fig. 2:**
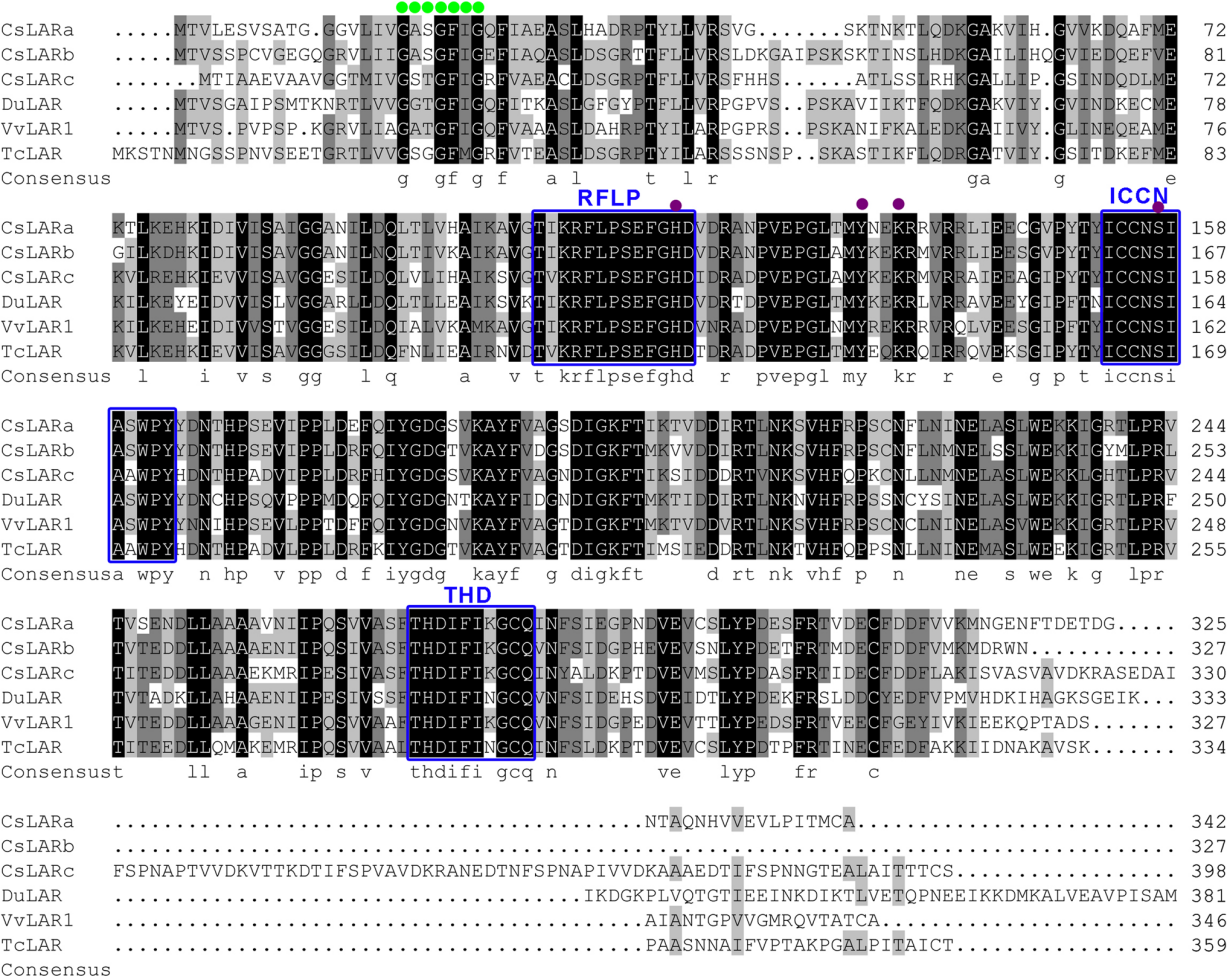
Sequence comparison of the LARs from the tea plant and other selected species. NADP-binding domain and substrate-binding sites are marked by green dots and pink dots, respectively, according to the scheme of VvLAR. LAR-specific motifs RFLP, ICCN and THD are marked by a blue box. Sequences are from *Theobroma cacao* (ADD51358), *Vitis vinifera* (AAZ82410) and *Desmodium uncinatum* (CAD79341). Identical amino acids are marked by white letters on a black background; conservative amino acids are marked by a dark gray background (similarity >75%); similar amino acids are indicated by black letters on a light gray background (similarity >50%); other amino acids are marked by black letters on a white background (similarity <50%). Sequence alignment was performed using the DNAMAN program

The amino acid sequences of the CsLARs were aligned with LAR proteins in other species using the DNAMAN program (Lynnon Corporation, San Ramon, CA, USA). The results showed that CsLARa had 71.88 and 54.41% amino acid sequence identity with CsLARb and CsLARc, respectively. The amino acid sequence alignment diagrams (Fig. 2) showed that the CsLARs have a putative glycine-rich NADP-binding domain (marked with a green dot) that is located in the N-terminal region, and several substrate binding sites (marked with pink dot) scattered in different regions were predicted based on the scheme of VvLAR (Mauge et al. 2010). Three LAR-specific amino acid motifs, RFLP, ICCN and THD (marked with a blue box), were consistent with other LARs (Hammerbacher et al. 2014).

### Evolutionary analysis of the plant LAR family

Two hundred and six candidate LAR sequences (containing duplicate sequences) were screened in different species using the CsLARs as seed sequences against the NCBI database with the blastP method (identity >51%). The sequences with less than 50% similarity to the CsLARs are IFR homologous sequences. To obtain as many LAR sequences of different species as possible, we also searched the transcriptomes of different species with the base sequences of the *CsLARs* to find sequences with high similarity (identity >65%). Then, we used the ORF finder software (https://www.ncbi.nlm.nih.gov/orffinder/) to find the open reading frames and translated them into amino acids. The functional LARs reported previously were constructed as models to test the candidate sequences (Suppl. Fig. S1). All LAR sequences have the LAR-specific motifs (RFLP, ICCN, and THD) and NADPH-binding site GXXGXXG located at the N-terminus. To illustrate this phenomenon more clearly, we chose a subset of these sequences for analysis in this paper.

**Fig. 3:**
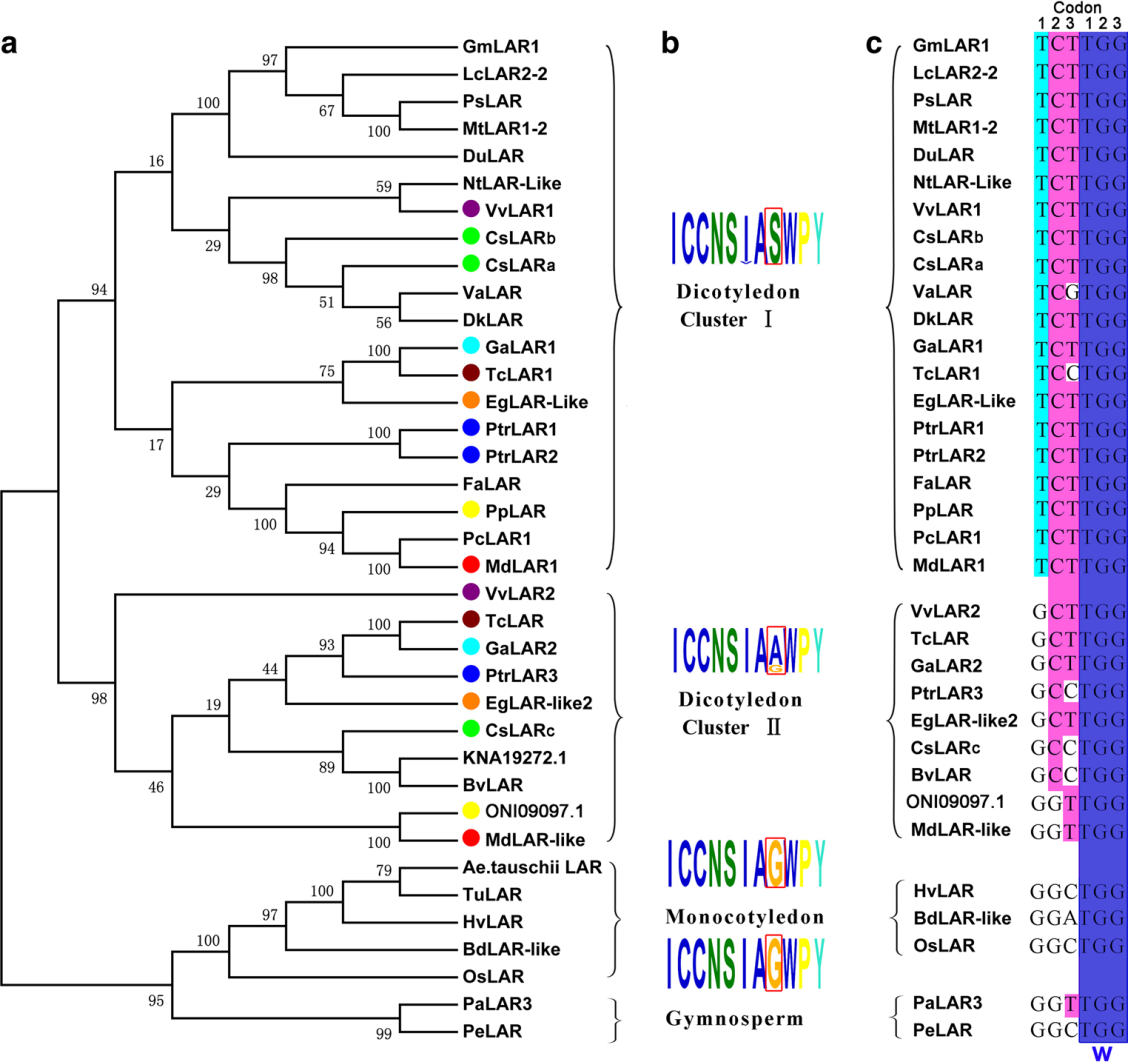
Phylogenetic relationships of LAR proteins from different species and a conserved motif and the corresponding codon analysis. **a** The phylogenetic tree of LAR proteins from the tea plant and other species. The phylogenetic analysis was performed using the neighbor-joining method with 1000 bootstrap replicates by MEGA version 5.0. The numbers indicate the confidence percentages. The accession numbers of the protein sequences obtained from GenBank are as follows: PaLAR3 (*Picea abies*, AMB51440.1); PeLAR (*Pinus taeda*, CAI56321.1); OsLAR (*Oryza sativa*, CAI56328); HvLAR (*Hordeum vulgare*, CAI56320); TuLAR (*Triticum urartu*, EMS45018.1); BdLAR-like (*Brachypodium distachyon,* XP_003561802.1); Ae.tauschii LAR (*Aegilops tauschii*, EMT15815.1); CsLARa (*Camellia sinensis,* KY615698), CsLARb (*Camellia sinensis*, KY615700), CsLARc (*Camellia sinensis*, KY615699); GaLAR1 (*Gossypium arboretum*, CAI56319); GaLAR2 (*Gossypium arboretum*, CAI56323.1); TcLAR1 (*Theobroma cacao*, EOY02147.1); TcLAR (*Theobroma cacao*, ADD51358); PtrLAR1 (*Populus tomentosa*, EEE89746); PtrLAR2 (*Populus tomentosa*, EEF01056); PtrLAR3 (*Populus trichocarpa*, EEF06163); VvLAR1 (*Vitis vinifera*, AAZ82410); VvLAR2 (*Vitis vinifera*, AAZ82411); BvLAR-like (*Beta vulgaris*, XP_010695625.1); MdLAR1 (*Malus domestica*, AFZ93007.1); MdLAR-Like (*Malus domestica*, XP_008356649.1); PpLAR (*Prunus persica*, EMJ23473.1); EgLAR-Like 2 (*Eucalyptus grandis*, KCW49054.1); EgLAR-Like (*Eucalyptus grandis*, XP_010044298.1); DuLAR (*Desmodium uncinatum*, CAD79341); GmLAR (*Glycine max*, AEM23933.1); LcLAR2-2 (*Lotus corniculatus*, ABC71329.1); MtLAR1-2 (*Medicago truncatula*, AES62081.1); PsLAR (*Pisum sativum*, AII26024.1); NtLAR-Like (*Nicotiana tomentosiformis*, XP_009614312.1); PcLAR1 (*Pyrus communis*, ABB77696.1); VaLAR (*Vaccinium ashei*, BAM42674.1); DkLAR (*Diospyros kaki*, ACI41981.1); and FaLAR (*Fragaria ananassa,* AFP99288.1); KNA19272.1 and ONI09097.1 were two hypothetical proteins selected from *Spinacia oleracea* and *Prunus persica*. **b** Three types of conserved ICCN motifs in LAR amino acid sequences. The amino acid residue in the red box was changeable during the evolutionary process. **c** The corresponding codons of the ICCN motif. The blue color indicates the codon of a conserved Trp (W), and the red color indicates the codon of the variable amino acid residue

A phylogenetic tree was constructed using the neighbor-joining method based on the above-mentioned amino acid sequences (Fig. 3a). The phylogenetic analysis showed that plant LARs could be grouped into two main clades. One clade included the dicotyledon group, and the other included the gymnosperm group and the monocotyledon group, which suggested that monocotyledon LARs were evolutionarily closer to gymnosperm LARs than to dicotyledon LARs.

The dicotyledonous LARs could be further clustered into two subgroups, which were defined as cluster I and cluster II. The LARs in *C. sinensis, V. vinifera*, *T. cacao*, *P. trichocarpa*, *Gossypium arboreum*, *Malus* × *domestica*, *Pyrus communis*, *Eucalyptus grandis* and *Prunus persica* were clustered into cluster I and cluster II. However, the LARs from Leguminosae, such as *Glycine max, Lotus corniculatus, Pisum sativum* and *M. truncatula*, constituted a monophyletic group that belonged to cluster I.

An amino acid residue varied in a conserved LAR-specific amino acid motif (ICCNSIAG/A/SWPY) (Fig. 3b). The sequence alignment of LARs in the phylogenetic tree showed that the eighth amino acid residue in the “ICCNSIAG/A/SWPY” motif in the gymnosperm and monocotyledon groups was a Gly residue, and Ser and Ala were in the same location in dicotyledonous cluster I and cluster II, respectively (Fig. 3b). Therefore, the gymnosperm and monocotyledon groups and dicotyledonous cluster I and cluster II could be named G-type LARs, S-type LARs and A-type LARs, respectively, based on this variable amino acid. The corresponding nucleotide sequence alignment showed that a transversion (G → T) and a transition (G → C) occurred in the first and second position of this codon, respectively (Fig. 3c). This finding suggested that the plant LAR family might have undergone a mutation in the eighth amino acid position in the conserved motif ICCN due to evolutionary pressures imposed by natural selection. According to the crystal structure of the VvLAR protein, the ICCN motif is located near the substrate binding site, suggesting this mutation may be of importance to the activity of the plant LARs (Mauge et al. 2010).

### Correlation between the content of the catechins and the transcription levels of the CsLARs in tea plants

Tea is rich in polyphenolic compounds, which mainly consist of six flavan-3-ols: EGCG, ECG, EGC, EC, GC and C. The contents of the six main catechins extracted from the bud, leaf, stem and young root of the tea plants were detected by HPLC (Cui et al. 2016). The results showed that the contents of the epicatechins (EGCG, ECG, EGC and EC) were much higher than those of the nonepicatechins (C and GC), and the contents of the galloylated catechins (EGCG and ECG) were much higher than those of the nongalloylated catechins (C, GC, EC, and EGC). EGCG and ECG were the predominant catechins in the leaves (Suppl. Fig. S2a). The accumulation of catechins was greater in the buds and younger leaves than in the mature leaves, stems and roots. However, no other catechins, except EC, were detected in the roots.

To investigate the transcription patterns of the *CsLARs* in different tea organs, quantitative real-time PCR (qRT-PCR) was performed. The results showed that the transcription profiles of *CsLARa* and *CsLARb* were similar, exhibiting relatively high levels in the tender shoots and leaves and low levels in the stems and roots (Suppl. Fig. S2b). *CsLARc* was preferentially expressed in the roots and moderately expressed in the leaves and stems; its expression level in the roots was twice as abundant as in the leaves. The transcription levels of the three *CsLARs* in the mature leaves were the lowest among all of the leaf organs, which was consistent with the catechin distribution profiles in the mature leaves.

A correlation analysis showed that the accumulation of the catechins was positively correlated with the expression of *CsLARa* and *CsLARb.* The transcriptional levels of *CsLARa* and *CsLARb* had the highest correlation with the accumulation pattern of EGC and the lowest correlation with the accumulation pattern of C; the corresponding correlation coefficients were 0.7323 and 0.3970 for *CsLARa* and 0.7816 and 0.6474 for *CsLARb*, respectively. The accumulation of the six catechins was negatively correlated with the expression of *CsLARc*.

### Functional verification of recombinant CsLARs expressed in *E. coli*

**Fig. 4:**
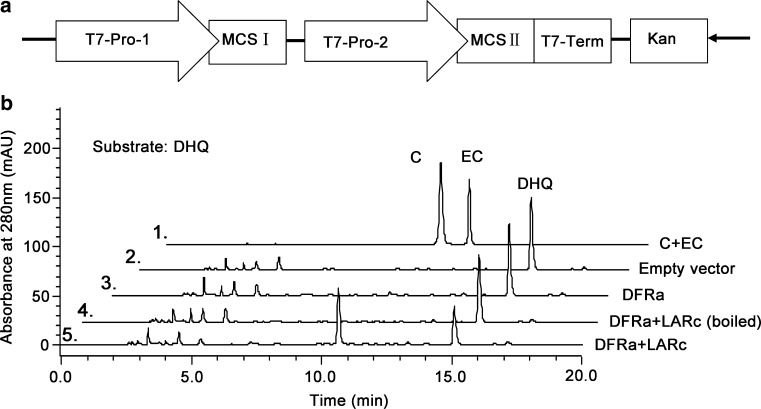
Identification of products from recombinant CsLARs in *E. coli*. **a** pRSF plasmid construct used for the co-expression of CsLARs and CsDFRa. **b** HPLC analysis of the product from the reaction of the recombinant proteins. Lines 1, 2, 3, 4 and 5 indicate the standards, empty vector control, CsDFRa alone reaction control, boiled enzyme control and CsDFRa + CsLARc assay, respectively

Leucoanthocyanins, which are the direct substrates of LARs, are unstable and not commercially available. Therefore, we cannot directly detect the catalytic activity of the CsLAR proteins alone. Thus, we used a strategy that included co-expressing CsDFRa (KY615690) and LARs in *E. coli* to verify the function of the CsLARs with DHQ, DHK and DHM as the substrates (Fig. 4a). CsDFRa can catalyze these substrates to produce leucoanthocyanins, which then serve as the substrates for the CsLARs. The results of the enzymatic assay showed that CsDFRa coupled with CsLARc could catalyze DHQ to produce the product catechin. No product was detected with the empty vector, DFRa alone or the boiled DFRa + CsLARs controls, which indicated that our strategy was feasible (Fig. 4b). The three CsLARs combined with DFRa could catalyze the dihydroflavonols (DHK, DHQ and DHM) and generate the corresponding (2R, 3S)-*trans*-flavan-3-ols (AFZ, C and GC), which were identified using UPLC coupled to a triple quadrupole mass spectrometer (QQQ-MS) (Suppl. Fig. S3).

### Functional verification of the CsLARs in *Arabidopsis*

In *Arabidopsis thaliana*, abundant PAs are present in the brown seed coat, including soluble and insoluble PAs, which mainly consist of an EC unit (Dixon et al. 2005). In addition, there is no LAR ortholog in the *A. thaliana* genome. Therefore, *A. thaliana* is a suitable model plant to use to verify whether LAR is involved in PAs biosynthesis.

**Fig. 5:**
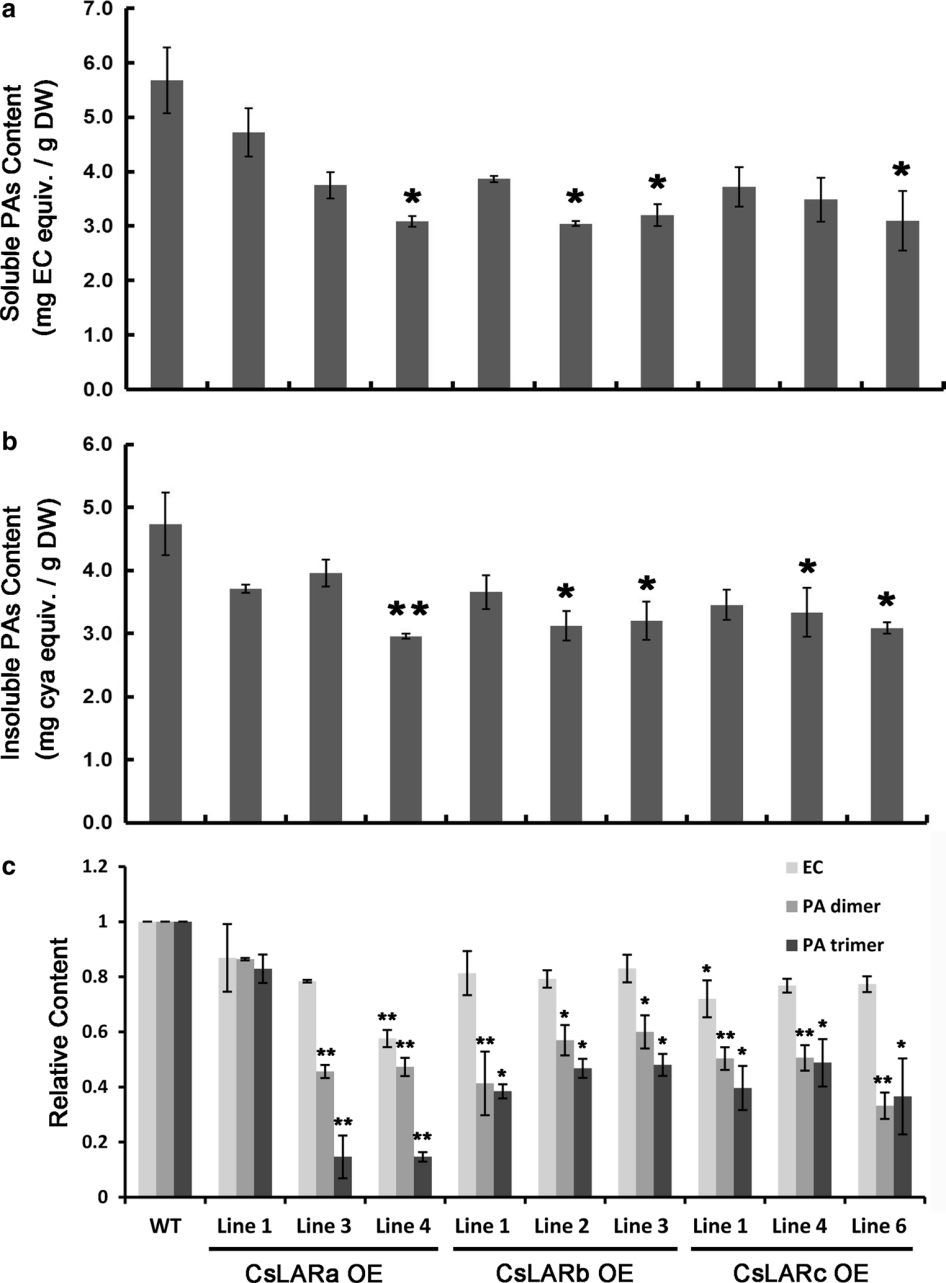
Overexpression of CsLARs in wild-type *A. thaliana.* **a** Soluble PAs content in WT and transgenic *A. thaliana* seeds. **b** Insoluble PAs content in WT and transgenic *A. thaliana* seeds. **c** The relative contents of EC, PA-dimers and PA-trimers in WT and transgenic *A. thaliana* seeds. The relative content is converted according to the peak area in a quantitative MRM model by UPLC–MS. All data are the means of three biological replicates, and the error bars represent the standard deviation of three replicates. The contents of EC, PA-dimers and PA-trimers from WT were set as 1.0. The *asterisks* indicate the significant level (*n* = 3, *P < 0.05, **P < 0.01) based on a Tukey’s honestly significant difference test

According to the phenotype of the transgenic *A. thaliana* overexpressing CsLARs, some lines showed a slight decrease in the brown color of the seed coat (data not shown). To detect the contents of the insoluble and soluble PAs in the seed coats, DMACA staining and an acid hydrolysis assay were conducted. The results showed that the contents of both insoluble and soluble PAs extracted from the seeds were reduced in the overexpressing CsLARs lines compared with WT (Fig. 5a, b). The UPLC–MRM analysis of the extracts showed that the contents of the EC-monomer (*m*/*z* 289.0), PA-dimer (*m*/*z* 577.0) and PA-trimer (*m*/*z* 865.0) were decreased in the *A. thaliana* overexpressing CsLARs (Fig. 5c). This reduction in the EC monomers and polymers was consistent with the observed decrease in the brown color of the seed coat. Although CsLARs catalyzed leucocyanidins to produce catechin in vitro, no catechin could be detected in any transgenic *A. thaliana* lines, which was inconsistent with the results of the recombinant CsLARs in *E. coli.*

Dixon and coworkers found that MtLAR could catalyze a PA extension unit, 4β-(*S*-cysteinyl)-epicatechin, to form EC, resulting in a decrease of PAs (Liu et al. 2016). The degree of decrease in the EC-monomer was less than that of the polymers in the *A. thaliana* overexpressing CsLARs (*m*/*z* 577.0, *m*/*z* 865.0), which could be explained by this theory (Fig. 5c).

### Functional verification of the CsLARs in tobacco

In contrast to *A. thaliana*, anthocyanins accumulate in the flowers of wild tobacco, and a slight accumulation of PAs was also detected due to the extremely low expression levels of the *ANR* and *LAR* genes. Thus, tobacco is a good model plant for studying the accumulation of anthocyanins and the biosynthesis of PAs.

**Fig. 6:**
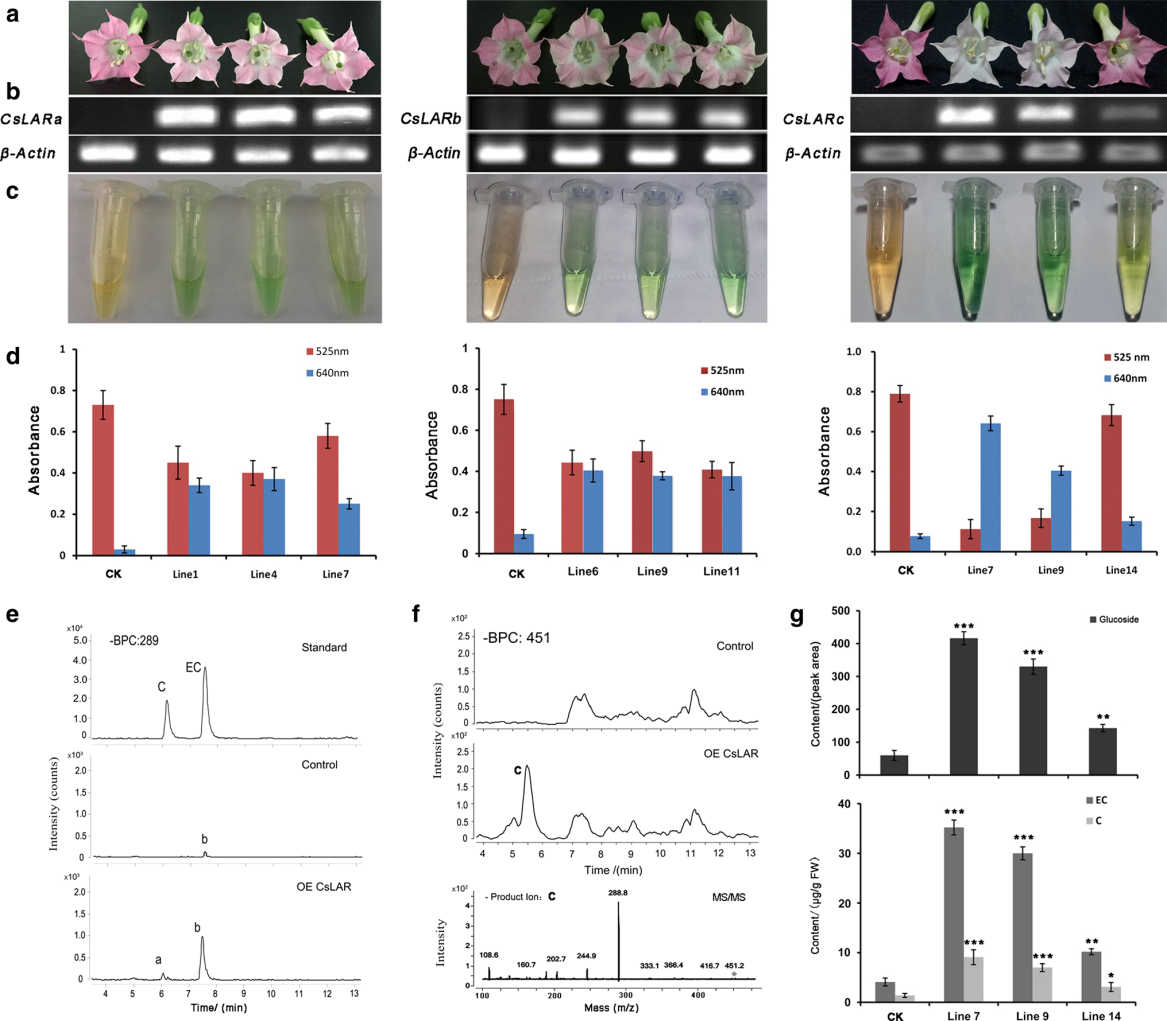
Overexpression of CsLARs in wild-type tobacco. **a** Phenotypes of empty vector control (CK) and CsLARs transgenic tobacco flowers. **b** Semi-quantitative RT-PCR analysis of the *CsLAR* and the housekeeping gene *NtActin* transcription levels in total RNA from the flowers. **c** DMACA staining of the extracts from transgenic tobacco flowers and CK. **d** The absorbance of the anthocyanins extracts and DMACA-reactive compounds at 525 and 640 nm, respectively. **e** Extraction ion chromatogram of products (*m*/*z* 289.0) accumulated in the transgenic tobacco flowers and CK. Authentic standard of catechin and epicatechin (top); products accumulated in the transgenic tobacco flowers (bottom) and CK (middle). **f** Extraction ion chromatogram of products (*m*/*z* 451.0) in the transgenic tobacco flowers (bottom) and CK (top). UPLC–MS/MS of peak c (*m*/*z* 451.0) is listed in the box. **g** The contents of glycoside, C and EC extracted from different transgenic lines of overexpressing CsLARc tobacco flowers and CK. The content of glycoside is indicated by the peak area as determined by UPLC–MRM-MS. The contents (mg/g, FW) of C and EC were quantified according to the peak area based on the standard C and EC. All data are the means of three biological replicates, and the error bars represent the standard deviation of three replicates. The asterisks indicate the significance level (*n* = 3, *P < 0.05, **P < 0.01, ***P < 0.001) based on a Tukey’s honestly significant difference test

To experimentally demonstrate the functions of the CsLARs, the open reading frames of the CsLARs were transferred into tobacco under the control of the *Cauliflower mosaic virus* 35S promoter. More than 30 transgenic T1 lines of CsLARa, CsLARb and CsLARc were generated for further research. The petals of flowers overexpressing CsLARs showed a lighter pink color compared to the controls (empty vector, CK). In addition, the intensity of the pink color was negatively correlated with the transcriptional abundance of the *CsLARs* (Fig. 6b). Among these lines, certain CsLARc transgenic lines with high transcription levels became almost white (Fig. 6a). The extracts of the transgenic tobacco flowers exhibited blue staining after reacting with the DMACA reagent, indicating that DMACA-reactive compounds were generated in the tobacco. The content of the DMACA-reactive compounds in the flower petals increased, and the content of anthocyanins decreased as the expression levels of the CsLARs increased (Fig. 6c, d).

To determine whether there were oligomeric/polymeric PAs in the extracts and the petal residues, a butanol/HCl hydrolysis assay was performed. No anthocyanin class material was generated after hydrolysis, indicating that there were no PAs in the transgenic tobacco (Suppl. Fig. S4). The content of the anthocyanin class material in the transgenic tobacco was lower than that in the control after the butanol/HCl hydrolysis, indicating that overexpression of the CsLARs caused a decrease in the PAs.

UPLC–MS/MS analysis showed that the DMACA-reactive compounds were the flavan-3-ol monomers EC and C (Fig. 6e). In addition, an EC (or C)–glycoside was also detected (Fig. 6f). The contents of EC, C and glycoside were positively correlated with the transcriptional abundance of the *CsLARs* (Fig. 6g). UPLC–MRM-MS results showed that the accumulation of EC, C and glycoside in the CsLARc line was 8.58, 6.42 and 6.89 times greater than that in the control, respectively (Table 1).

**Table 1:** Levels of selected flavonoid compounds in various transgenic and control tobacco. The data were determined by UPLC–MS. The content (mg/g, FW) of C, EC and procyanidin B2 were quantified according to the peak area based on the standards. All data are the mean values of three biological replicates

Compound name	Levels (μg/g, FW)			
	CK (control)	CsLARc	CsLARc + AtPAP1	AtPAP1
EC (C)–glycoside*	60.3	415.6	1615.5	65.4
Catechin	1.19	7.64	38.08	1.46
Epicatechin	3.47	29.79	128.72	4.74
PA-dimers (B2)	0.74	0.32	0.88	1.09
PA-trimers*	20.31	8.92	10.52	25.1

The asterisk represents the substance for which we do not have the standards. So, the values represent the peak area

In summary, the transgenic CsLARs tobacco flowers showed a visibly decreased color and produced a series of DMACA-reactive flavan-3-ol monomers containing mainly EC, C and glycoside. However, no PA oligomer/polymer was detected in the CsLAR transgenic tobacco flowers.

### The interrelationships between the LAR and anthocyanidin pathways

**Fig. 7:**
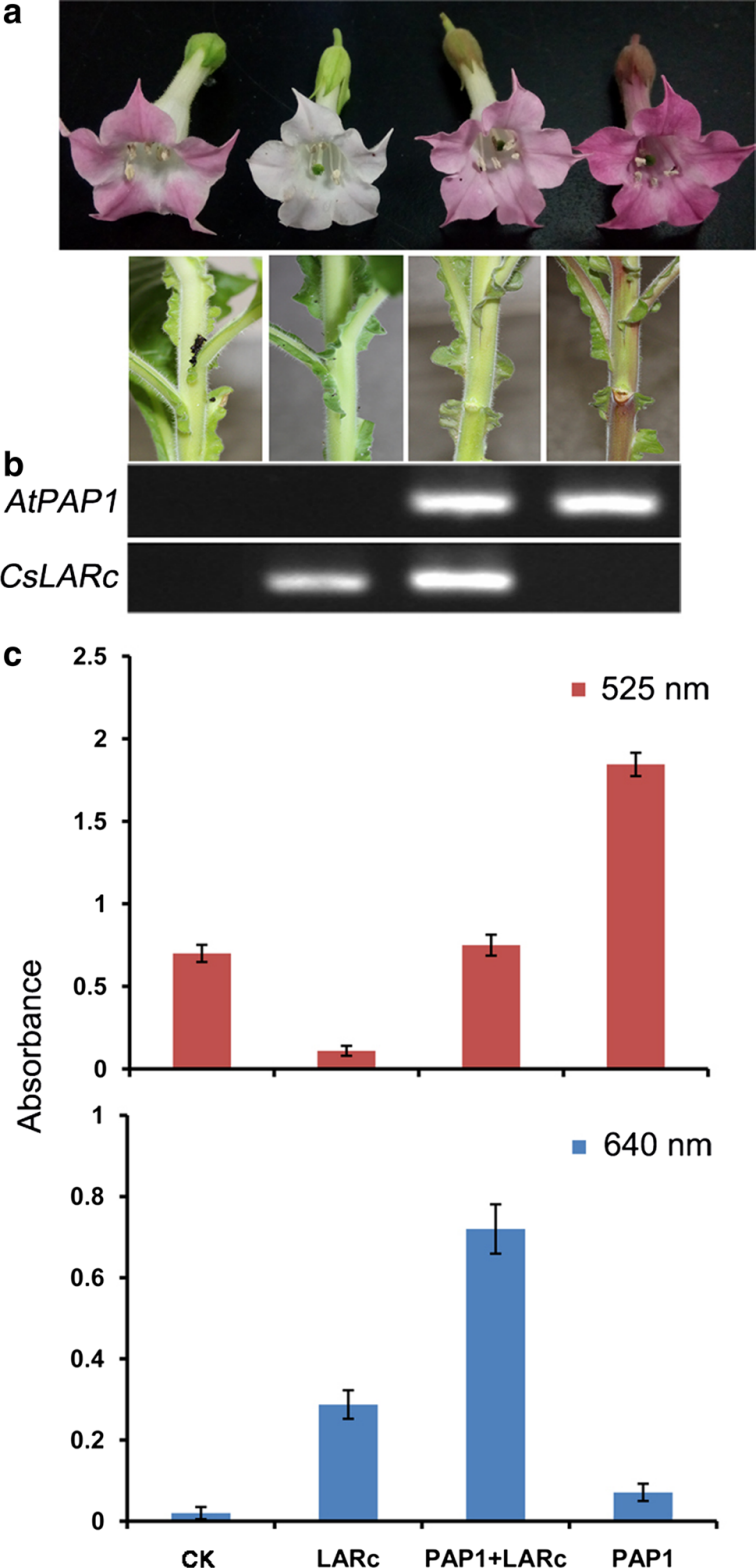
Hybridization of the CsLARc transgenic tobacco and AtPAP1 transgenic tobacco. **a** Phenotypes of CK, CsLARc, AtPAP1 and CsLARc + AtPAP1 transgenic tobacco. **b** Semi-quantitative RT-PCR analysis of the *CsLARc* and *AtPAP1* transcription levels in total RNA from the flowers shown in **a**. **c** The absorbance of the anthocyanins extracts and DMACA-reactive compounds at 525 and 640 nm, respectively

To determine the interrelationships between the LAR and anthocyanidin pathways, a hybridization experiment was performed using CsLARc and AtPAP1 transgenic tobacco that highly accumulated anthocyanins in the leaves and flowers. Through cross-pollination hybridization, several herbicide-resistant CsLARc + AtPAP1 transgenic tobacco lines were generated. The transgenic lines with high transcription levels of both *CsLARc* and *AtPAP1* were selected for further analysis along with AtPAP1, CsLARc and CK tobacco lines as controls. Regarding the phenotype, the CsLARc + PAP1 transgenic tobacco exhibited a visibly reduced pink pigmentation in the leaves, stems and flowers compared with the AtPAP1 controls, but the pigmentation was still darker than that in the CsLARc-expressing and CK tobacco lines (Fig. 7a, b). The measurement of the anthocyanins extracted from the flowers at 525 nm was consistent with the observed phenotype (Fig. 7c). DMACA-reactive compounds were generated only in the CsLARc + AtPAP1 and CsLARc transgenic tobacco, and the absorbance value of CsLARc + PAP1 was double that of the CsLARc transgenic tobacco. The UPLC–MRM-MS analysis indicated that the accumulation of EC, C and glycoside in the CsLARc + AtPAP1 line was 4.32, 4.98 and 3.88 times greater than that in the CsLARc line and 27.15, 26.08 and 24.70 times greater than that in the AtPAP1 line, respectively (Table 1). The contents of the PA-dimers and -trimers in the flower extracts of the CsLARc + AtPAP1 line were 0.80 and 0.42-fold greater than those in the AtPAP1 line, respectively. Similarly, no insoluble PAs were detected in the residues of any transgenic lines by the butanol/HCl hydrolysis assay. Overexpression of the LARs reduced the content of anthocyanins and accumulated EC, C and glycoside in the tobacco, but it did not promote the biosynthesis of PAs.

### The coefficients of LAR and ANR in the PA biosynthetic pathway

Certain R2R3 MYB transcription factors, such as AtTT2, VvMYBPA1 and MtMYB14, have been reported to regulate the biosynthesis of PAs by regulating *ANR* and *LAR* (Nesi et al. 2001; Bogs et al. 2007; Liu et al. 2014). According to the conserved amino acid sequence, a homologous TT2 R2R3 MYB transcription factor, named CsMYB5b (KY827397), in the tea plant was selected and overexpressed in tobacco to examine the interrelationships between LAR and ANR.

**Fig. 8:**
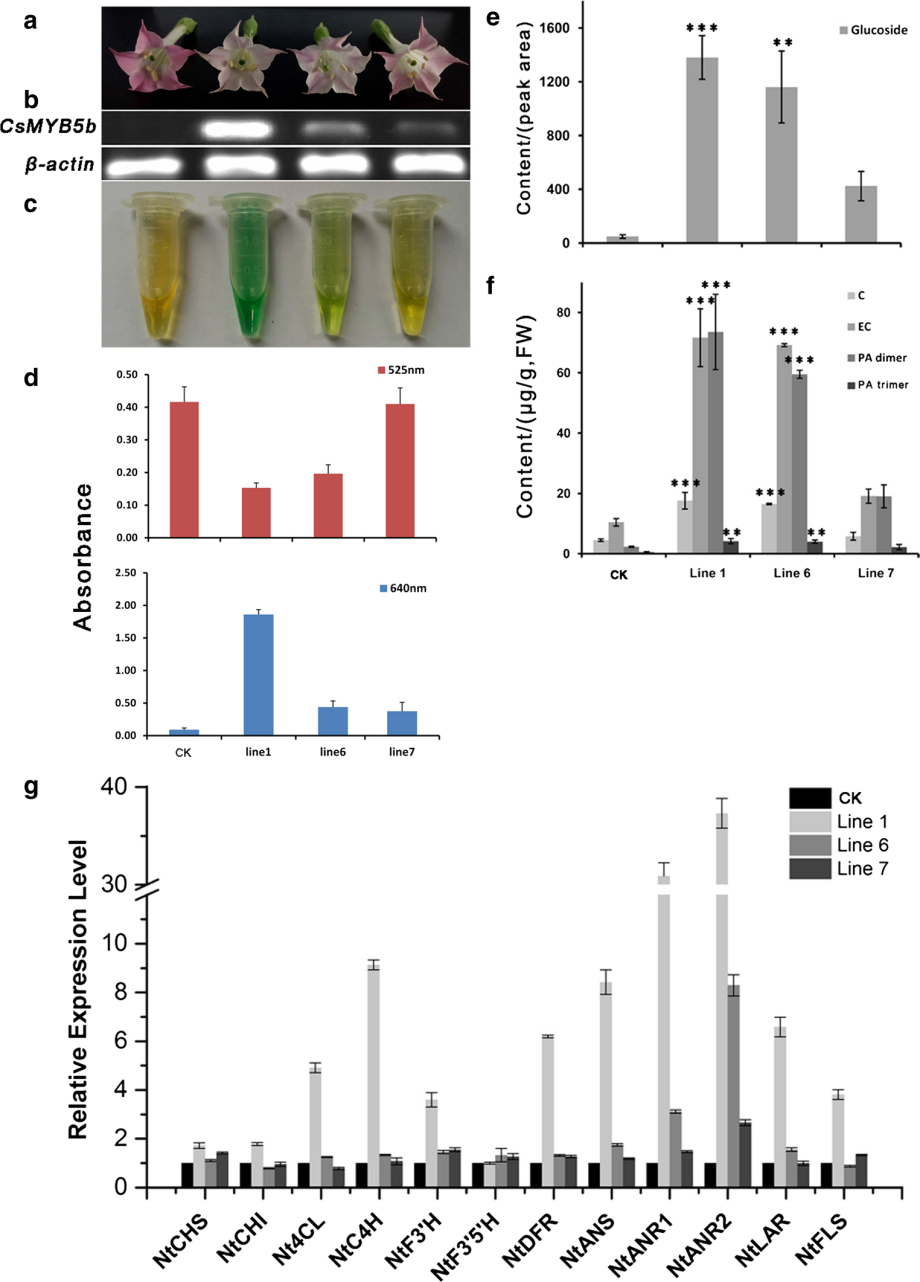
Overexpression of CsMYB5b in wild-type tobacco. **a** Phenotypes of CK and CsMYB5b transgenic tobacco flowers. **b** Semi-quantitative RT-PCR analysis of the *CsMYB5b* and the housekeeping gene *NtActin* transcription levels in total RNA from the flowers shown in **a**. **c** DMACA staining of the extracts from transgenic tobacco flowers and controls. **d** The absorbance of the anthocyanins extracts and DMACA-reactive compounds at 525 and 640 nm, respectively. **e** The content (peak area) of glycosides extracted from different transgenic lines of overexpressing CsMYB5b tobacco flowers and CK. **f** The contents (μg/g, FW) of C, EC, PA-dimer and PA-trimer extracted from different transgenic lines of overexpressing CsMYB5b tobacco flowers and CK. The PA-trimer was also quantified according to the standard procyanidin B2. All data are the means of three biological replicates, and the error bars represent the standard deviation of three replicates. The *asterisks* indicate the significant level (*n* = 3, *P < 0.05, **P < 0.01, ***P < 0.001) based on a Tukey’s honestly significant difference test. **g** Expression profiles of structural genes in flowers of different transgenic CsMYB5b tobacco lines

The phenotypes of transgenic CsMYB5b-expressing tobacco were consistent with those of transgenic CsLAR tobacco (Fig. 8a). The petals of flowers overexpressing CsMYB5b also exhibited lighter pink color, and the intensity of the pink color was negatively correlated with the transcription levels of CsMYB5b. Simultaneously, the content of the DMACA-reactive compounds was increased with the increase in the transcription levels of *CsMYB5b* (Fig. 8b–d).

Unlike the CsLARs transgenic tobacco, EC, C and glucoside were detected, but additionally, PA polymers (*m*/*z* 577.0 and *m*/*z* 865.0) accumulated in the overexpressing CsMYB5b tobacco, and their accumulation was positively correlated with the expression levels of *CsMYB5b* (Fig. 8e, f). Among them, the content of EC was three times greater than C, and the PA-dimer was ten times greater than the PA-trimer. Butanol/HCl hydrolysis experiments also showed that the overexpressing CsMYB5b tobacco accumulated a massive amount of PA polymers (including soluble and insoluble PAs), which were never detected in the tobacco overexpressing CsLARs (Suppl. Fig. S3). qRT-PCR analysis revealed that *NtANR1*, *NtANR2* and *NtLAR* were the main up-regulated genes in all of the transgenic lines (Fig. 8g). *Nt4CL*, *NtC4H*, *NtF3′H* and *NtFLS*, which are involved in the flavonoid pathway, were up-regulated (more than twofold) in CsMYB5b transgenic tobacco. Unlike *NtANR1*, *NtANR2* and *NtLAR*, *NtC4H, NtDFR* and *NtANS* only exhibited higher expression levels in transgenic Line 1 (Fig. 8g). The results implied that the biosynthesis of PAs might require the co-expression of *NtANRs* and *NtLAR.* Expression of LAR alone only promoted the biosynthesis of flavan-3-ol monomers. The primers used in qRT-PCR are listed in Suppl. Table S2.

## Discussion

### Evolutionary analysis of LARs in plants

Flavonoids evolution has been well discussed from the phylogenetic, chemotaxonomic, enzymatic, and molecular perspectives (Harborne 1989; Stafford 1991; Koes et al. 1994; Grotewold 2006). The enormous variation in the types of flavonoids produced by different plants may be due to gene duplication and divergence, pleiotropy and loss-of-function mutations that occurred during adaptive evolutionary changes (Grotewold 2006). For example, the emergence of certain new enzymes or functions resulted from initial gene duplication, such as *CHS*, *F3H* and *CHI* (Verwoert et al. 1992; Jez et al. 2000; Winkel-Shirley 2001). The biosynthetic branch pathway of PAs can be traced back to ferns, which are the most primitive vascular plants (Grotewold 2006). The flavanols, which are precursors of PA biosynthesis, are widely distributed in gymnosperms and angiosperms and have also been detected in ferns (Stafford 1991; Peng et al. 2012). Many experiments have shown that the accumulation of phenolic compounds is closely related to plant resistance against biotic and abiotic stresses, which contributes to the adaptation of plants to environmental stresses (Dixon et al. 2005; Fornalé et al. 2014). An independent evolution of a specific F3′5′H in the Asteraceae family, which provides the basis for their purple flower color, likely reflects the adaptive value of attraction for insect pollination (Seitz et al. 2006).

The LARs and ANRs are known to be responsible for the biosynthesis of flavan-3-ols (Tanner et al. 2003; Xie et al. 2004). As shown in the phylogenetic tree (Fig. 3), the LARs are widely distributed from gymnosperms to angiosperms, although an LAR homolog has not been found in ferns. This finding is consistent with the evolutionary analysis of the ANR homologs, which are present in gymnosperms and dicotyledonous and monocotyledonous species (Peng et al. 2012).

The dicotyledonous LARs could be further clustered into two subgroups, i.e., cluster I and cluster II. Nevertheless, the LARs from Leguminosae, such as *G. max*, *L. corniculatus*, *P. sativum* and *M. truncatula,* constituted a monophyletic group that belonged to cluster I. Similarly, a previously reported phylogenetic tree of the *DFR* genes also showed that the *DFRs* of leguminous plants constituted a monophyletic group (Shimada et al. 2005). The evolutionary significance of this phenomenon is currently unclear.

LARs in plants were classified as G-type LARs (gymnosperms and monocotyledons groups), A-type LARs (dicotyledonous cluster II) and S-type LARs (dicotyledonous cluster I) based on a variable amino acid in the conserved ICCN motif. Further analysis revealed that a transversion (G → T) and a transition (G → C) mutation occurred in the first and second positions of this codon, respectively. Though the ICCN motif is located near the substrate-binding site according to VvLAR protein crystal structure, we did not see an obvious difference among the three CsLARs (CsLARa, b belong to S-type LARs and CsLARc belongs to A-type LARs) in either in vivo or in vitro experiments. A previously reported PaLAR3 (a G-type LAR, from gymnosperm *Picea abies*) also had the same function of catalyzing leucocyanidin to produce catechin in vitro (Hammerbacher et al. 2014). It suggests that the effect of this polymorphism is likely to be quite subtle.

Protein evolution has been studied for many years (Echave and Wilke 2016). Studying whether the mutation in the eighth amino acid in the ICCN motif affects the protein function, evolutionary rate, kinetic stability, native state stability and active structure stability of the LAR protein is warranted.

### The predicted function of LAR in plants

To date, all LARs in different plants have been reported to have the catalytic products AFZ, C and GC in vitro with leucoanthocyanidins as substrates (Stafford and Lester 1984; Kristiansen 1986; Tanner et al. 2003; Pang et al. 2007). However, transgenic plants constitutively overexpressing LARs resulted in different characteristics, which raised new questions regarding the real function of LARs in vivo. In transgenic DuLAR tobacco and white clover plants, no detectable levels of catechin or polymer were found (Tanner et al. 2003). The overexpression of *MtLAR* in tobacco reduced the content of anthocyanins in the flower petals, but no desired catechin or increased PAs were detected in the flowers or leaves (Pang et al. 2007). The transgenic TcLAR tobacco showed decreased amount of anthocyanidins and unexpectedly generated both EC and C monomers in the tobacco flowers (Liu et al. 2013). The ectopic expression of CsLAR in the tea cultivar TRI2043 in *M. truncatula* hairy roots and a type of purple tobacco accumulated a massive amount of anthocyanins and EC, respectively (Pang et al. 2013). In our experiments, the coupled DFR + LAR assays indicated that a single product of C was generated and DHQ served as the substrate. The overexpression of CsLARs in tobacco and the hybridization assay of CsLARc and AtPAP1 both indicated that EC was the main product, along with the synthesis of minus C. In addition, no increased polymers were detected in the extracts or residues. In contrast, the content of the PAs in the tobacco overexpressing CsLARs was reduced (Suppl. Fig. S3). This finding is somewhat consistent with the findings of a decrease in the PAs content in flowers from transgenic tobacco overexpressing MdLAR1 or an increase in the insoluble PAs in the seeds of the *lar M. truncatula* mutants (Liao et al. 2014; Liu et al. 2016). Therefore, we hypothesize that other substrates of LAR may exist in tobacco in addition to leucocyanidins.

This supposition has recently been explored. Dixon and coworkers found that MtLAR could catalyze a new substrate, 4β-(*S*-cysteinyl)-epicatechin, to form EC in vitro (Liu et al. 2016). Excessive 4β-(*S*-cysteinyl)-epicatechin, which is a carbocation form, played an important role in non-enzymatic polymerization and served as a PA extension unit in *M. truncatula.* Yet, the source of 4β-(*S*-cysteinyl)-epicatechin in *M. truncatula* is unknown. The biosynthesis of carbocation is particularly important, and a further understanding of this process will be helpful for more rationally explaining the true function of LAR in vivo. In addition, more direct genetic evidence, such as mutations in LAR, need to be obtained to elucidate the real function of LAR in vivo.

#### *Author contribution statement*

LG and TX conceived and designed the experiment. PW performed most of experiments and wrote the paper. LZ and TL cultivated the transgenic tobacco. XJ analyzed the data of LC–MS. XD extracted and determined the metabolites of transgenic tobacco. LX and DX extracted and determined the metabolites of transgenic *Arabidopsis thaliana*. YL and ML modified the language of the paper. All authors read and approved the final version of the manuscript.

## Electronic supplementary material

Below is the link to the electronic supplementary material.

Suppl. Fig. S1 Sequence alignment of the LAR proteins from plants. NADP-binding domain and substrate binding sites are marked by green dots and pink dots, respectively, according to the scheme of VvLAR. LAR-specific motifs RFLP, ICCN, and THD are marked by a blue box. Asterisks indicate three types of changeable amino acids in the ICCN motif, which is marked by a red box. Identical amino acids are marked by white letters on a black background; conservative amino acids are marked by a dark gray background (similarity > 75%); similar amino acids are indicated by black letters on a light gray background (similarity > 50%); and other amino acids are marked by black letters on a white background (similarity < 50%). Sequence alignment was performed using the DNAMAN program. These amino acid sequences are consistent with the sequences in the phylogenetic tree (Fig. 2) (JPEG 4622 kb)

Suppl. Fig. S2 The accumulation profiles of catechins and the expression patterns of the three *CsLAR* genes in various organs. a The quantitative analysis of C, GC, EC, EGC, ECG, and EGCG in different organs. b The expression profiles of *CsLARa*, *CsLARb*, and *CsLARc* in different organs. All data are the means of three biological replicates, and the error bars represent the standard deviation of three replicates. The different letters (a, b, c, d…) indicate the significant level at *P* < 0.05 based on a Tukey’s honestly significant difference test (JPEG 1186 kb)

Suppl. Fig. S3 Identification of the products from recombinant the CsLARs with different substrates in *E. coli*. a, b, c indicate the HPLC chromatograms (Left) and MS/MS analysis (Right) of the products from CsDFRa + CsLARs reactions with DHK, DHQ and DHM as substrates. Arabic numerals 1 and 2 indicate the products and substrates, respectively. Note, the standard afzelechin in the DHK reaction was not available (JPEG 3831 kb)

Suppl. Fig. S4 Butanol/HCl hydrolysis assay of polymerized catechins from transgenic tobacco flowers and controls. a Butanol/HCl hydrolysis of soluble polymerized catechins. b The content (μg/g, FW) of polymerized catechins in different transgenic lines of tobacco. c Butanol/HCl hydrolysis of insoluble PAs. d The content (μg/g, FW) of insoluble PAs in different transgenic lines of tobacco. All data are the means of three biological replicates, and the error bars represent the standard deviation of three replicates. The asterisks indicate the significant level (*n* = 3, ***P < 0.001) based on a Tukey’s honestly significant difference test (JPEG 635 kb)

Supplementary material 5 (DOC 175 kb)

Supplementary material 6 (DOCX 30 kb)

## Abbreviations

ANR: Anthocyanidin reductase
C: Catechin
DFR: Dihydroflavonol 4-reductase
DMACA: Dimethylaminocinnamaldehyde
EC: Epicatechin
GC: Gallocatechin
LAR: Leucoanthocyanidin reductase
