# Supplementary figures and images for: Evolutionary and functional characterization of leucoanthocyanidin reductases from Camellia sinensis
Source: Planta. 2017 Sep 8;247(1):139–54. doi: 10.1007/s00425-017-2771-z (PMC5756577; doi:10.1007/s00425-017-2771-z)

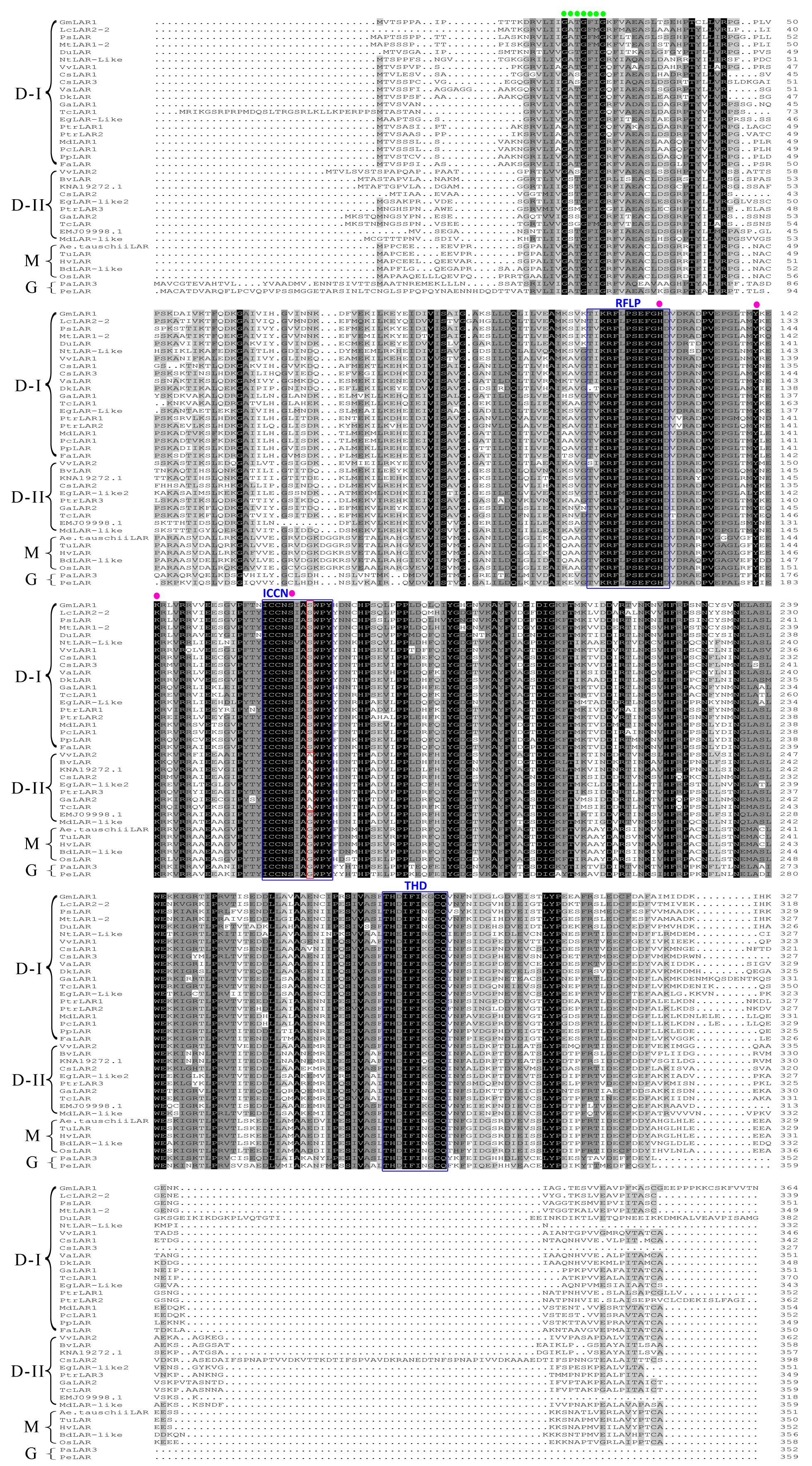

Supplement: Supplementary file 1 — Suppl. Fig. S1 Sequence alignment of the LAR proteins from plants. NADP-binding domain and substrate binding sites are marked by green dots and pink dots, respectively, according to the scheme of VvLAR. LAR-specific motifs RFLP, ICCN, and THD are marked by a blue box. Asterisks indicate three types of changeable amino acids in the ICCN motif, which is marked by a red box. Identical amino acids are marked by white letters on a black background; conservative amino acids are marked by a dark gray background (similarity > 75%); similar amino acids are indicated by black letters on a light gray background (similarity > 50%); and other amino acids are marked by black letters on a white background (similarity < 50%). Sequence alignment was performed using the DNAMAN program. These amino acid sequences are consistent with the sequences in the phylogenetic tree (Fig. 2) (JPEG 4622 kb) [file 425_2017_2771_MOESM1_ESM.jpg]

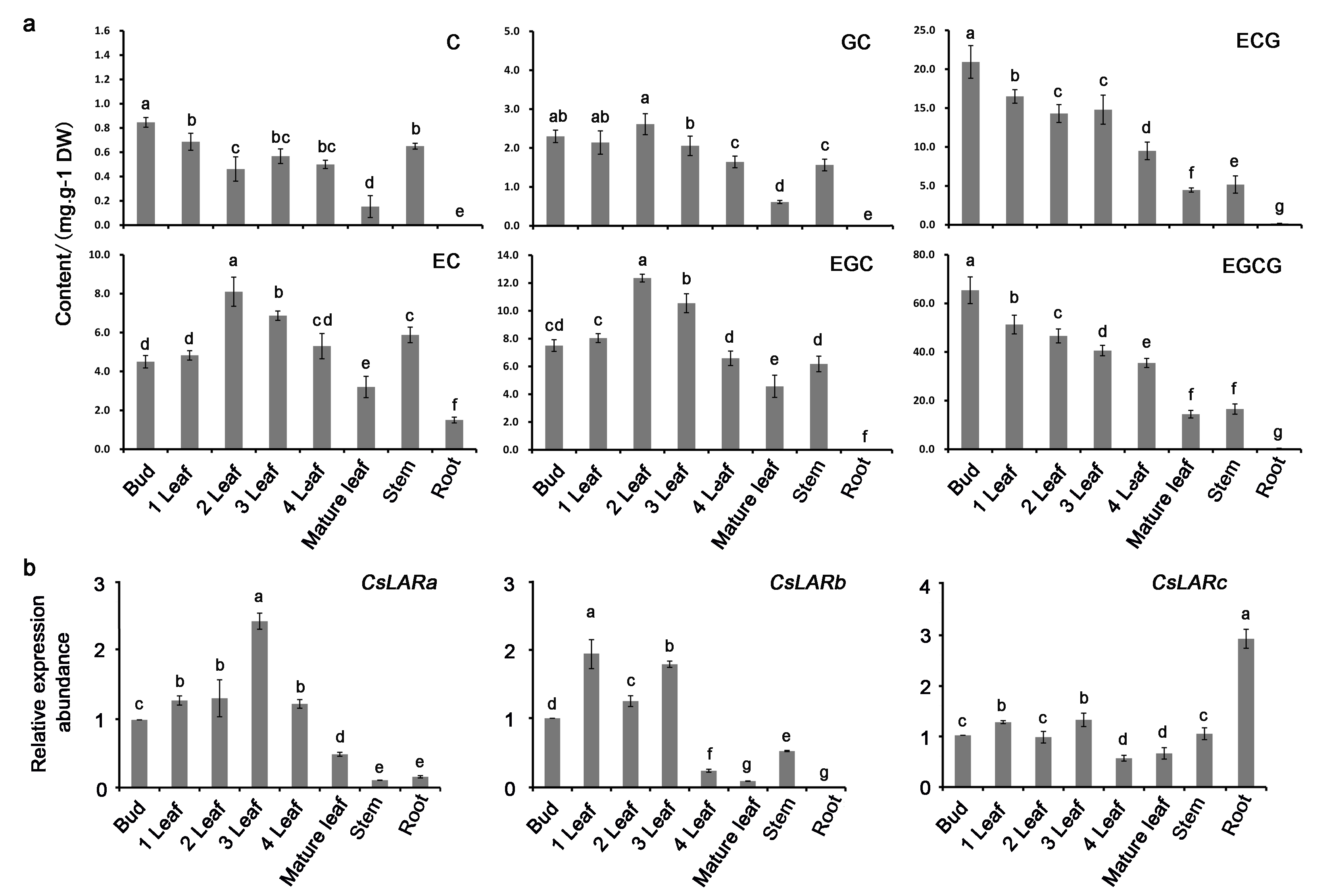

Supplement: Supplementary file 2 — Suppl. Fig. S2 The accumulation profiles of catechins and the expression patterns of the three CsLAR genes in various organs. a The quantitative analysis of C, GC, EC, EGC, ECG, and EGCG in different organs. b The expression profiles of CsLARa, CsLARb, and CsLARc in different organs. All data are the means of three biological replicates, and the error bars represent the standard deviation of three replicates. The different letters (a, b, c, d…) indicate the significant level at P < 0.05 based on a Tukey’s honestly significant difference test (JPEG 1186 kb) [file 425_2017_2771_MOESM2_ESM.jpg]

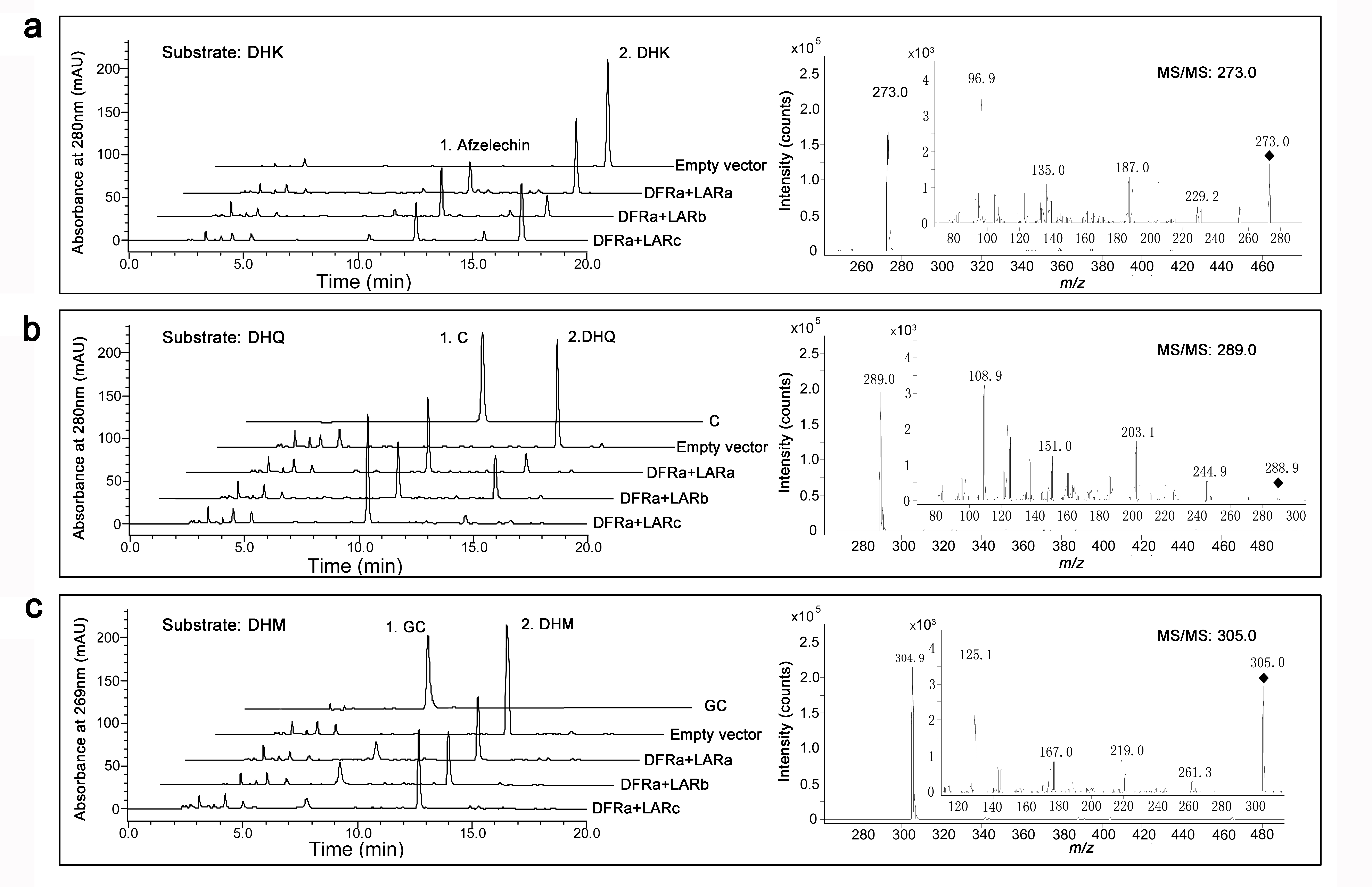

Supplement: Supplementary file 3 — Suppl. Fig. S3 Identification of the products from recombinant the CsLARs with different substrates in E. coli. a, b, c indicate the HPLC chromatograms (Left) and MS/MS analysis (Right) of the products from CsDFRa + CsLARs reactions with DHK, DHQ and DHM as substrates. Arabic numerals 1 and 2 indicate the products and substrates, respectively. Note, the standard afzelechin in the DHK reaction was not available (JPEG 3831 kb) [file 425_2017_2771_MOESM3_ESM.jpg]

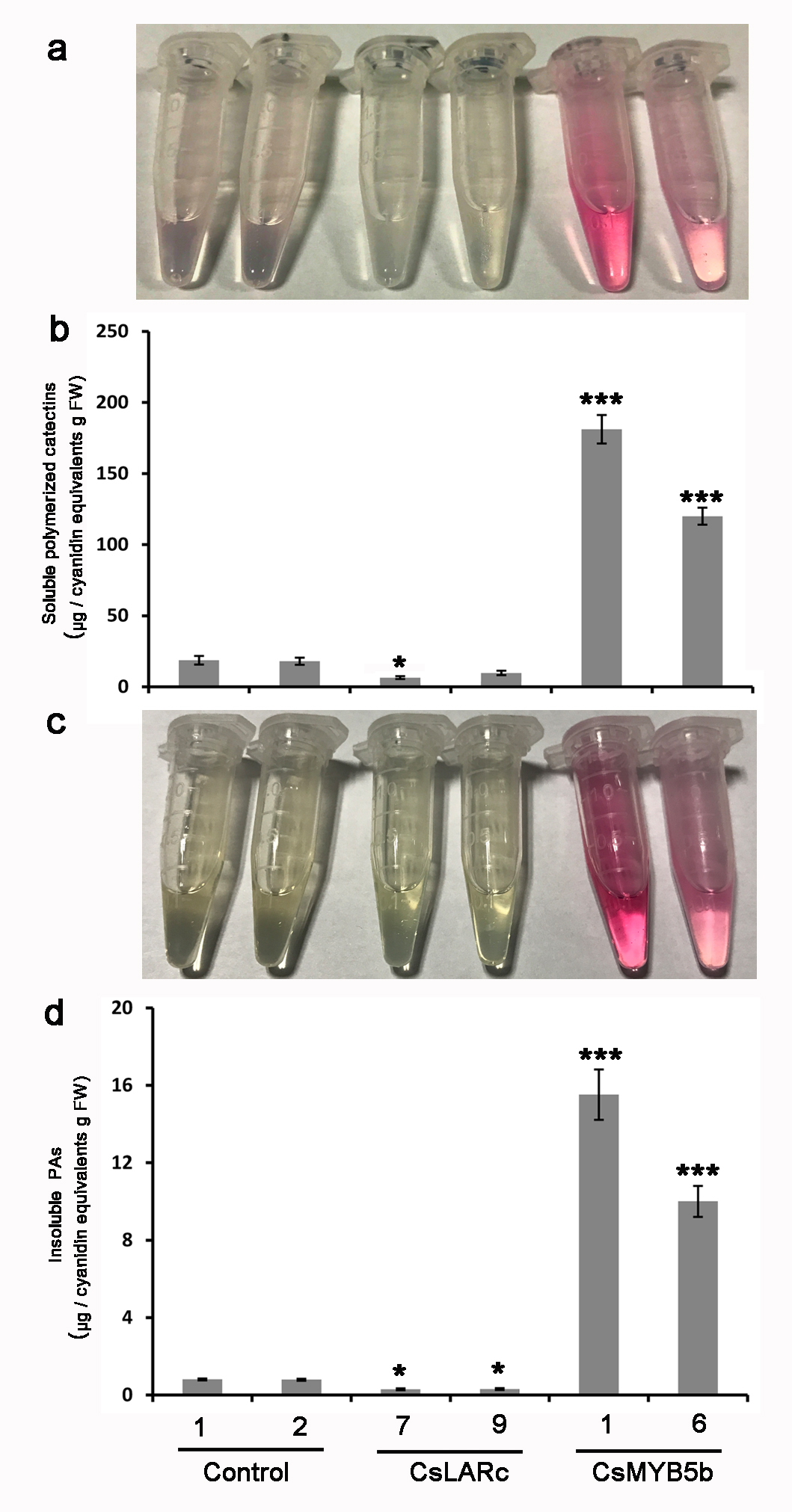

Supplement: Supplementary file 4 — Suppl. Fig. S4 Butanol/HCl hydrolysis assay of polymerized catechins from transgenic tobacco flowers and controls. a Butanol/HCl hydrolysis of soluble polymerized catechins. b The content (μg/g, FW) of polymerized catechins in different transgenic lines of tobacco. c Butanol/HCl hydrolysis of insoluble PAs. d The content (μg/g, FW) of insoluble PAs in different transgenic lines of tobacco. All data are the means of three biological replicates, and the error bars represent the standard deviation of three replicates. The asterisks indicate the significant level (n = 3, ***P < 0.001) based on a Tukey’s honestly significant difference test (JPEG 635 kb) [file 425_2017_2771_MOESM4_ESM.jpg]
