# Supplementary material for: Evolutionary and functional characterization of leucoanthocyanidin reductases from Camellia sinensis
Source: Planta. 2017 Sep 8;247(1):139–54. doi: 10.1007/s00425-017-2771-z (PMC5756577; doi:10.1007/s00425-017-2771-z)
Supplement: Supplementary file 5 — Supplementary material 5 (DOC 175 kb) [file 425_2017_2771_MOESM5_ESM.doc]

**Suppl. Table S1** Sequences of primers used for DNA cloning, construction of protein expression vectors, quantitative RT-PCR and construction of heterologous expression vectors.

| Purpose | Gene name | primer name | | primer sequence (5'-3') |
| --- | --- | --- | --- | --- |
| DNA cloning | *CsLARa* | | UPM: | CTAATACGACTCACTATAGGGCAAGCAGTGGTATCAACGCAGAGTACATGGGG |
|  |  |  | NUP： | AAGCAGTGGTATCAACGCAGAGT |
|  |  |  | 3′race-outer | GAAGTGTGCAGCCTCTACCCTGATG |
|  |  |  | 3′race-inner | CACTGCCCAGAATCATGTTGTTGAAG |
|  |  |  | 5′race-outer | CGAGAAGGTACGTAGGCCGATCAGC |
|  |  |  | 5′race-inner | CGGTTGCGGACACAGATTCCAACAC |
|  |  |  | ORF-F: | **ATG**ACTGTGTTGGAATCTG |
|  |  |  | ORF-R: | TCAAGCACACATTGTGATG |
|  | *CsLARc* | | 3′race-outer | GTTGCCAGATAAACTATGCCCTCG |
|  |  |  | 3′race-inner | GGTAGTTGACAAAAGGGCAAGTGG |
|  |  |  | 5′race-outer | GGTGTAGGGGATCCCAGCCTC |
|  |  |  | 5′race-inner | CAACCTCAATCTTATGTTCCCTC |
|  |  |  | ORF-F: | **ATG**ACTATAGCAGCAHAAG |
|  |  |  | ORF-R: | TCATGAACAAGTGGTGGTG |
|  | *CsLARb* | | 3′race-outer | CAATTGATGGTCCACATGAAGTTGAAG |
|  |  |  | 3′race-inner | GATTTTGTTATGAAGATGGATCGATGG |
|  |  |  | 5′race-outer | CAAGGCTGGCTTGAGCAATGAACTC |
|  |  |  | 5′race-inner | GACCGTGTCAAGCCCTTGTGTTGGAG |
|  |  |  | ORF-F: | **ATG**ACCGTGTCAAGCCC |
|  |  |  | ORF-R: | TTAATTCCATCGATCCATC |
| Construction of protein expression vectors | *CsDFRa+CsLARa* | | *CsDFRa*-SacⅠ-F | GCGAGCTCG**ATG**AAAGACTCTGTTGC |
|  |  |  | *CsDFRa*-SalⅠ-R | GAGTCGACTTAAACCTTGTT GCC |
|  |  |  | *CsLARa*-NdeⅠ-F | GCCATATG**ATG**ACTGTGTTGGAATCTG |
|  |  |  | *CsLARa*-XhoⅠ-R | CCGCTCGAGAGCACACATTGTGATGG |
|  | *CsDFRa+CsLARc* | | *CsDFRa*-NdeⅠ-F | GCCATATG**ATG**AAAGACTCTGTTGC |
|  |  |  | *CsDFRa*-XhoⅠ-R | CCGCTCGAGTTAAACCTTGTTGCC |
|  |  |  | *CsLARc*-EcoRⅠ-F | GGAATTC**ATG**ACTATAGCAGCAGAAG |
|  |  |  | *CsLARc*-PstⅠ-R | GCACTGCAGTGCTCATGAACAAGTGG |
|  | *CsDFRa+CsLARb* | | *CsDFRa*-SacⅠ-F | GCGAGCTCG**ATG**AA AGACTCTGTTGC |
|  |  |  | *CsDFRa*-SalⅠ-R | GAGTCGACTTAAACCTTGTTGCC |
|  |  |  | *CsLARb*- NdeⅠ-F | GCCATATG**ATG**ACCGTGTCAAGCCC |
|  |  |  | *CsLARb*-XhoⅠ-R | CCGCTCGAGATTCCATCGATCCATC |
| Quantitative RT-PCR | *CsGAPDH* | | *CsGAPDH-*qRT*-*F  *CsGAPDH-*qRT*-*R | TTGGCATCGTTGAGGGTCT  CAGTGGGAACACGGAAAGC |
|  | *CsLARa* | | *CsLARa*-qRT-F | AAAAGAGGAGGGTGCGG |
|  | *CsLARb* | | *CsLARa*-qRT-R  *CsLARb*-qRT-F | GGAACTCATCCAAAGGGGG  TCAGAGTTTGGACATGACGTGG |
|  |  | | *CsLARb*-qRT-R | CTGACTCCTCTATCAACCTCCG |
|  | *CsLARc* | | *CsLARc*-qRT-F | GCCCCACCTTTCTTCTCGT |
|  | *CsLARa* | | *CsLARc*-qRT-R  *CsLARa+attb-*F | CCAACGGCTGAGATTACAACC  GGGGACAAGTTTGTACAAAAAAGCAGGCT**ATG**ACTGTGTTGGAATCTG |
| Construction of heterologous expression vectors | *CsLARb*  *CsLARc*  *CsMYB5b* | | *CsLARa+attb-*R  *CsLARb+attb-*F  *CsLARb+attb-*R  *CsLARc+attb-*F  *CsLARc+attb-*R  *CsMYB5b+attb-*F  *CsMYB5b+attb-*R | GGGGACCACTTTGTACAAGAAAGCTGGGTTCAAGCACACATTGTGATGG  GGGGACAAGTTTGTACAAAAAAGCAGGCT**ATG**ACCGTGTCAAGCCCTTG  GGGGACCACTTTGTACAAGAAAGCTGGGTTTAATTCCATCGATCCATCTTC  GGGGACAAGTTTGTACAAAAAAGCAGGCT**ATG**ACTATAGCAGCAGAAG  GGGGACCACTTTGTACAAGAAAGCTGGGTTCATGAACAAGTGGTGGTGATAG  GGGGACAAGTTTGTACAAAAAAGCAGGCT**ATG**GGTAGGAGACCTTGTTG  GGGGACCACTTTGTACAAGAAAGCTGGGTTTATTGATCTTGAAGCCAATC |

Note：The restriction enzymes sites in the primers were underlined.
