# Supplementary material for: Evolutionary and functional characterization of leucoanthocyanidin reductases from Camellia sinensis
Source: Planta. 2017 Sep 8;247(1):139–54. doi: 10.1007/s00425-017-2771-z (PMC5756577; doi:10.1007/s00425-017-2771-z)
Supplement: Supplementary file 6 — Supplementary material 6 (DOCX 30 kb) [file 425_2017_2771_MOESM6_ESM.docx]

**Suppl. Table S2** Sequences of primers used for quantitative RT-PCR in transgenic tobaccos.

| Purpose | Gene name | | Primer name | | Primer sequence (5'-3') |
| --- | --- | --- | --- | --- | --- |
|  | | *NtCHS*  *NtCHI*  *Nt4CL*  *NtC4H* | *NtCHS*-qRT- F  *NtCHS-*qRT- R  *NtCHI*-qRT- F  *NtCHI* -qRT- R  *Nt4CL-*qRT- F  *Nt4CL*-qRT- R  *NtC4H*-qRT- F  *NtC4H*-qRT- R | TGCTAAGCGAATACGGGAAC  AACAGAAACACTGCGGAGGA  GCAGTCTCTTCAAAGTACACGCC  GAGAGCAATGGAGTCTGTTACCGT  TGGCTACATTGATGATGACGAC  CCACTGGAACTTCTCCTGCTT  GGGTATCGCCGAGTTAGTCAA  ATCACAGCCTGAAGGTATGGAA | |
| Quantitative  RT-PCR | | *NtF3’H*  *NtF3’5’H*  *NtDFR*  *NtANS*  *NtANR1*  *NtANR2*  *NtLAR*  *NtFLS* | *NtF3’H-*qRT- F  *NtF3’H* -qRT- R  *NtF3’5’H-*qRT- F  *NtF3’5’H* -qRT- R  *NtDFR*-qRT- F  *NtDFR* -qRT- R  *NtANS-*qRT- F  *NtANS* -qRT- R  *NtANR1*-qRT- F  *NtANR1*-qRT- R  *NtANR2-*qRT- F  *NtANR2*-qRT- R  *NtLAR-*qRT- F  *NtLAR* -qRT- R  *NtFLS*-qRT- F  *NtFLS*-qRT- R | TTGTCCCGCAATGACTTACG  GCATAGTAGGTAGGCGAGGTG  TCCCTAGAATCTCGAACGAACC  TCCCACTCAAGAACCTTTCAGG  GCGAAAGGGAGGTATATGTGCTC  TGCTTGTCCCTCGGTACTCAGTA  ACTACTACCCCAAATGTCCCCAAC  CCGTTACCCACTGTCCTTCATAGA  CTTGAAGGGTATGCAGATGTT  GCAGAGCAAACATATCGTCCAG  AGCGTGTCGTTTTGACCTCATC  CCAATTAGACTCATCCACGACG  GCAGCAGAAGACTATAGAACTGTG  CATGTGTTAGAGCTGCAACTACAC  TGAAGGGAAAAGGGGTTGG  ACTCCTCATTTGCTTCCCTGTAG | |
|  | | *NtActin* | *NtActin*-qRT- F  *NtActin*-qRT- R | TAGAAACCCCAAGTACCCTCG  TGCTTTCTTCGTCCCATCAG | |
